# Comparing AI and human moral reasoning: context-sensitive patterns beyond utilitarian bias

**DOI:** 10.3389/frai.2025.1710410

**Published:** 2026-01-12

**Authors:** Elyas Barabadi, Zahra Fotuhabadi, Amanollah Arghavan, James R Booth

**Affiliations:** 1 Department of Foreign Languages, University of Bojnord, Bojnord, Iran; 2 Faculty of Letters and Humanities, Ferdowsi University of Mashhad, Mashhad, Iran; 3 Department of Psychology and Human Development, Vanderbilt University, Nashville, TN, United States

**Keywords:** artificial intelligence, deontology, foreign language effect, large language models, moral judgment, utilitarian

## Abstract

**Introduction:**

Decision-making supported by intelligent systems is being increasingly deployed in ethically sensitive domains. As a result, it is of considerable importance to understand the patterns of moral judgments generated by large language models (LLMs).

**Methods:**

To this end, the current research systematically investigates how two prominent LLMs (i.e., ChatGPT and Claude Sonnet) respond to 12 moral scenarios previously administered to human participants (first language and second language users). The primary purpose was to examine whether the responses generated by LLMs align with either deontological or utilitarian orientations. Our secondary aim was to compare response patterns of these two models to those of human respondents in previous studies.

**Results:**

Contrary to prevailing assumptions regarding the utilitarian tendency of LLMs, the findings revealed subtle response distributions of moral choice that are context-sensitive. Specifically, both models alternated between deontological and utilitarian judgments, depending on the scenario-specific features.

**Discussion:**

These output patterns reflect complex moral trade-offs and may play a significant role in shaping societal trust and acceptance of AI systems in morally sensitive domains.

## Introduction

Artificial intelligence (AI) has become an increasingly tangible presence, transforming many aspects of people’s lives. As an assistant, AI can help individuals in various tasks, such as decision-making (e.g., Duan et al., 2019; Xiong et al., 2022). However, for people to rely on AI in such decisions, there should be some level of trust, which is not easily established. Past research studies demonstrate that people’s perception of AI is that it does not involve emotion and contextual understanding of a scenario or a situation. According to Lee’s (2018) research, people viewed AI algorithmic outputs as less reliable and fair compared to human decisions. Similarly, Shank et al. (2019) found that interactions with human-like AI systems can trigger intense feelings such as surprise and confusion during interactions. That is, people experience these emotions because they struggle with viewing AI as either a programmed machine or a human-like entity with emotions and intentions. Research indicates that people view AI systems as impersonal and dehumanized, but there is minimal research investigating differences between output patterns of AI systems and humans’ decision-making through utilitarian and deontological lenses during classic and realistic moral dilemmas. Recently, however, a growing body of research (see Almeida et al., 2024; Garcia et al., 2024; Sachdeva and Van Nuenen, 2025) has examined the overall alignment between Large Language Models (LLMs) output patterns with human data. Their findings suggest that despite some similarities between LLMs’ and humans’ responses to moral dilemmas, there are still some systematic differences between them, with some LLMs showing more alignment with human beings. People often see AI as producing utilitarian choices. Yet research on whether human participants using their first or second language make different or similar moral judgments compared to AI-generated choices remains minimal. This study intends to fill existing research gaps by systematically comparing generated choices from two LLMs (ChatGPT 4.0 and Claude Sonnet) with those made by human participants through carefully constructed moral dilemmas that were previously used in human research delivered in their first or second languages.

### Artificial intelligence and morality

AI is increasingly becoming an acceptable substitute for human beings in performing some tasks whose outputs have moral consequences, like automated car and cancer diagnosis (Waytz et al., 2014; Wilson et al., 2022). Research has recently investigated how humans morally perceive and react to such algorithmic outputs and observable system behaviors of AI-based machines (Giroux et al., 2022). For example, research shows that in real-life situations, where people interact with AI systems, human agents are judged as more wrong, more blameworthy, and more intentional than AI agents, although both agents perform the same harmful action (Wilson et al., 2022). Moreover, when it comes to how people talk about AI’s wrongdoing, people tend to use words such as “behave,” “learn,” “intend,” and “function.” In fact, people do not treat AI as a true moral agents, and they shift the blame to humans who designed it. This is in line with what Marchesi et al. (2019) refers to as mechanistic explanations. In other words, people describe what a system does in terms of mechanisms and functioning, not in terms of inner intentions, motives, or having a mind like a human.

Obviously, this is because AI is primarily viewed as a tool, not a moral agent. In line with this view, Myers and Everett (2025) invented and attributed the term ‘artificial moral advisors’ (AMAs) to AI systems that can assist humans in moral decision-making. Through their training data, these systems can analyze moral dilemmas and provide recommendations based on ethical theories and principles. Drawing on human beings’ mind perception studies, Zhang et al. (2022) also came to the conclusion that, compared to human beings, people tend to attribute higher competence but lower warmth to AI systems. Due to such differential perceptions between humans and AIs, people tend not to favor morally related outcomes supported by AI-based systems, partly because they believe that these systems do not take into account the unique characteristics of individuals (see Longoni et al., 2019). On the other hand, there is also evidence that disagrees with this view. For instance, in the classic trolley dilemma, Krügel et al.’s (2023) study suggests that ChatGPT’s responses varied based on how the dilemma was framed. Their findings suggest that this LLM’s output is more context-sensitive rather than purely utilitarian.

In moral psychology, human decision-making has been extensively studied through a deontological-utilitarian framework. However, there is limited understanding concerning AI-supported choices. Previous studies have only investigated human perception (Bigman and Gray, 2018; Rom et al., 2017; Zhang et al., 2022), ignoring the actual AI behavior. The dominant finding in the perception-focused literature is that AI systems lack empathy, are emotionally detached, and operate in a purely logical manner. With these qualities, people assume these systems prioritize outcomes over moral rules (Rom et al., 2017). Research suggests that LLMs’ responses are more rigid or predictable compared to humans’ responses. This contrast is perhaps due to their training data, which leads to unexpected responses (Takemoto, 2024). Such misalignment between human participants and LLM-generated outputs was also observed in some recent research (see Garcia et al., 2024; Sachdeva and Van Nuenen, 2025), highlighting the inherent complexity of moral decision-making.

Given the fact that people’s trust in AI is heavily shaped by these perceptions, particularly the assumption that AI-supported decisions are purely analytical, there is a higher probability that such views are biased (Magni et al., 2023). Because AI systems are being deployed in almost every human domain, making them an integral part of our lives and society, there is a need to understand where AI-supported choices lie on the utilitarian-deontology spectrum by examining the responses they would make if given the same set of moral dilemmas given to human participants. This will provide more evidence indicating whether AI-generated responses show utilitarian patterns associated with ‘cognitive’ decision-making, or whether, in specific situations, their response patterns approximate deontological judgments, in line with human affective decision makers. Taken together, we aim to understand whether the patterns of AI-supported choices in moral dilemmas are emergent and contingent, as human moral judgment appears to be, or relatively fixed and predetermined, as claimed by most extant literature.

### Humans’ moral judgment and foreign language effect

Imagine that you are a surgeon, and you have a patient who is in coma with 50% chance of survival. Would you sacrifice this patient to use his or her organs to save five other patients who are in desperate need of organ transplants? In the moral-psychology literature, if you agree to do this, it is typically interpreted as a utilitarian judgment, otherwise your choice is often taken to indicate a deontological one. People’s moral tendencies generally hinge upon a host of contextual and individual factors, including culture, religion, age, gender, and language, among others (Antón et al., 2020; Geipel et al., 2015). Over the past decade, the linguistic context in relation to decision-making in general (Díaz-Lago and Matute, 2018; Costa et al., 2014), and moral decision-making in particular (Barabadi et al., 2021), has been studied by numerous scholars. It has been established that when people use a foreign language, not only do they favor utilitarian moral decisions, but they are also less likely to fall prey to various cognitive biases, which is known as the foreign language effect (FLE) (Keysar et al., 2012).

A set of studies have suggested the dual system theory (Greene et al., 2008; Kahneman, 2003; Stanovich and West, 2000) may help explain the foreign language effect (see Hayakawa et al., 2017). According to this theory, human cognition processes information in two modes. System 1 usually operates in a way that our intuitions and reflexes guide our behavior, most of which is emotional, whereas System 2 is analytical and more effortful, requiring people to get involved in deliberation (Greene, 2014; Kahneman, 2003). As such, it is possible that since the linguistic repertoire of L2 does not carry the same emotional load and intensity as that of the first language, humans may be less emotionally involved and hence do not act based on heuristics and gut feeling (Evans, 2008; Kahneman, 2003, 2011). That is, when people make decisions in a native language, they are more likely to rely on intuitive, automatic processes (i.e., gut feelings), while use of a foreign language lowers emotional reactivity (Harris et al., 2003; Hayakawa et al., 2017) and may be linked to an increase in psychological distance (Trope and Liberman, 2010).

Alternatively, some researchers using dual-process theory to account for FLE believe that the disfluency in L2 compared to L1 makes individuals think about something in a more critical manner, leading to a more comprehensive examination of various aspects of that situation and hence coming to a utilitarian decision. Another factor that may play a key role is language-dependent memory. Emotions and experiences are often encoded in the language in which they occur, making them more accessible when the same language is used at retrieval (Marian and Neisser, 2000; Marian and Kaushanskaya, 2004; Schrauf and Rubin, 2000). That is, use of a foreign language may weaken the activation of certain mental constructs, such as cultural, religious, and emotional responses, that are tied to the native language. This line of research suggests an important role for language-specific memory and cultural accessibility in making moral judgments. The intricate and complex relationship between linguistic context (e.g., use of a foreign language) and moral judgment has led some researchers to suggest some task-specific and situation-specific justifications for when, where, and why using a second language might influence moral decisions (Geipel et al., 2015). Arguably, certain mental constructs, such as stereotypes, which have been shaped by years of cultural learning in a native language context, may exert less influence when using a foreign language (Geipel et al., 2015).

Taken together, the extant literature on foreign language use and AIs in relation to moral judgment suggests some overlap regarding the mechanisms that account for how L2 users make moral decisions and how AI systems generate responses in moral dilemmas. Both AI-based systems and second language users, whose responses may involve emotional distancing, may favor utilitarian-type responses, without considering the specific context of the moral dilemma. As such, we believe that the direct comparison of LLM responses to moral dilemmas to those of humans, including both first- and second-language users, can shed light on the moral response profiles of LLMs, as well as on the nature of the foreign language effect on such judgments. Such an awareness can inform evaluations of the role of AI-based systems in society, given their increasing integration into our daily life.

In examining this role more closely, Bajpai et al.’s (2024) study evaluated the apparent moral reasoning performance of chatbots that utilize LLMs, indicating that LLM-based chatbots have not yet reached human-like moral reasoning performance. These models also were reported to reflect a bias for individualistic moral foundations. Cheung et al. (2025), in contrast, found that LLM-generated responses were more altruistic than humans’ responses in collective action problems, while showing a stronger omission bias, favoring inaction over action. Similarly, Lei et al.’s (2024) study found that response patterns produced by ChatGPT-4o demonstrated a stronger belief in fairness and justice, consistent with its behavioral outcomes, whereas human beliefs show a wider range of fluctuations. Despite an enormous body of research on moral issues associated with AI, particularly how people trust it and accept it, previous studies have not directly compared LLMs’ responses to the same moral dilemmas responded to by human participants across first and second languages. The current study is an attempt to fill this gap by comparing the responses of two LLMs (namely, ChatGPT and Sonnet) to a set of moral dilemmas to those of human beings gathered in previous studies among first and second language users. More specifically, we aim to answer the following research question:

1. Do LLMs exhibit context-sensitive moral output patterns similar to humans, and if so, under what conditions?

## Methods

### Participants

Our human participants were Iranian Persian-English bilinguals (N = 1,675). The vast majority of them were self-identified as Muslims. In this study, the human data reflect a relatively homogeneous cultural and religious context, which enabled us to conduct a robust comparison with LLMs’ responses. However, it should be noted that human participants do not belong to this study, and we only used their data for the purpose of comparison.

### Moral dilemmas

To analyze and compare moral decision-making between humans and LLMs, we selected scenarios from the previous studies in which human participants provided answers to moral dilemmas in their L1 and L2 (see Table 1). These scenarios were selected from the following studies: Barabadi et al. (2021), Barabadi et al. (2022), Rahmani Tabar et al. (n.d.), and Barabadi et al. (n.d.). Below, we provide comprehensive descriptions of the moral dilemmas.

**Table 1:** Overview of previous human moral-judgment studies and scenarios used for LLM comparison.

Previous studies	Participants No		Age range	Languages used for scenarios			12 Scenarios used	Question/Prompt
	Female	Male						
				L1	L2	L2		
Barabadi et al. (2021)	192	132	18–24	Persian	English	Arabic	Classic trolley problem	Choose one of the following: A: the push option, B: the switch option, C: taking no action.
Barabadi et al. (2023)	351	143	18–48	Persian	English	N/A	COVID-19 dilemma (4 versions based on CNI model)	Is it acceptable in this case to remove your own son from ventilator? Yes No
Rahmani Tabar et al. (n.d.)	187	67	18–32	Persian	English	N/A	Six realistic scenarios: Ventilator; Car Crash; Company; Endangered Zoo Animals; ICU; Torture	Three choices whose morality was rated on a scale from 1 to 7. 1. Direct option (utilitarian), 2. Indirect option 3. Do nothing (inaction or omission).
Barabadi et al. (n.d.)	391	219	18–39	Persian	English	Arabic	Four scenarios: Wallet, Resume, Illegal Lunch, and Footbridge dilemma	Yes and No response format. For example, Do you lie on a resume to make it more convincing for getting a job?
	Total Female: 1121	Total Male: 561						

Source. Data compiled from Barabadi et al. (2021, 2023), Barabadi et al. (n.d.), and Rahmani Tabar et al. (n.d.).

Given the aim of this study, it was necessary to adopt a deontological-utilitarian framework since this framework was used in all previous studies from which we drew participants’ responses.

In the *Trolley Dilemma*, participants must decide whether to push a large stranger off a footbridge to stop a trolley, saving five workers at the cost of the stranger’s life, flip a switch to divert the trolley to a track where one worker will die to save three others, or do nothing and allow the workers to die. The response format is yes/no.

In the *COVID-19 Scenarios*, as a hospital director, participants are asked to make difficult decisions regarding their son and newly arrived COVID-19 patients. In one scenario, participants must decide whether to remove their son from a ventilator to save five newly arrived COVID-19 patients, knowing the son will die but the patients’ lives will be saved. In another scenario, they must decide whether to remove their son from a ventilator to save five newly arrived COVID-19 patients, knowing that the patients will survive but experience painful lung inflammation. In a third scenario, participants must decide whether to transfer their severely sick son to a more equipped hospital, risking infection of nurses for whom COVID-19 would not be fatal, knowing the son will die if not transferred. Finally, in the fourth scenario, participants must decide whether to transfer their severely sick son to a more equipped hospital, risking infection of nurses with medical conditions who could die from the virus. All scenarios are presented with a yes/no response format.

In the *Ventilator scenario*, participants, as the director of a hospital, must decide whether to remove a young man from a ventilator to save five COVID-19 patients, reduce the oxygen supply for the young man to save the patients indirectly, or do nothing. The responses are measured on a Likert scale, ranging from 1 (not at all moral) to 7 (very moral).

In the *Car Crash scenario*, participants must decide whether to swerve off the road to avoid hitting a mother and her children, risking running over an elderly woman, hitting the car in front of them, causing the driver’s death, or doing nothing. The responses are measured on a Likert scale, ranging from 1 (not at all moral) to 7 (very moral).

In the *Company scenario*, participants must decide whether to hand over an employee to smugglers, knowing they will die during the journey, shoot the employee to save themselves and others, or do nothing and risk being killed by rebels. The responses are measured on a Likert scale, ranging from 1 (not at all moral) to 7 (very moral).

In the *Endangered Zoo Animals scenario*, participants must decide whether to shoot the infected animals to save others, poison the parasites at the cost of five species becoming extinct, or do nothing. The responses are measured on a Likert scale, ranging from 1 (not at all moral) to 7 (very moral).

In the *ICU scenario*, participants must decide whether to use the organs of a comatose patient to save five others, transfer five injured patients to the ICU at the cost of one elderly patient’s life, or do nothing. The responses are measured on a Likert scale, ranging from 1 (not at all moral) to 7 (very moral).

In the *Torture scenario*, participants must decide whether to torture a criminal to obtain information about abducted children, release poisonous gas to extract the same information, or do nothing. The responses are measured on a Likert scale, ranging from 1 (not at all moral) to 7 (very moral).

Additionally, from another under-review study (Barabadi et al., 2021), we extracted a set of four scenarios, including the classic Footbridge dilemma used in our previous study, along with the Wallet, Resume, and Illegal Lunch dilemmas. In the *Footbridge dilemma*, participants must decide whether to push a large stranger off a footbridge to stop a trolley, saving five workers at the cost of the stranger’s life, or do nothing. In the *Wallet dilemma*, participants are asked if they would keep money found in a lost wallet to pay for their own urgent expenses. The *Resume dilemma* asks participants whether they would lie on a resume to make it more convincing for getting a job. Lastly, in the *Illegal Lunch dilemma*, participants must decide whether to meet with a judge socially to gain favor and help win a case. All scenarios are presented in a yes/no response format.

### LLMs selection and their training data

#### ChatGPT

ChatGPT is a type of AI designed for conversation created by OpenAI (OpenAI, 2023). More specifically, it uses different techniques, such as deep learning, to produce human-like texts. In particular, because of its transformer architecture, it has a strong performance at language-related tasks, including translation and summarization (Belatrix, 2024). Functionally, its main purpose is to generate responses which are logical, context-relevant, and appear natural to the reader. To achieve this, it is trained through analysis of a massive amount of text collected from the internet (Flensted, 2024). Because of its diverse training data, it can produce texts in many languages, such as Spanish, French, Chinese, and so forth. Part of its pre-training also involves guessing the next word in a sequence. In this setup, it does so by using preceding and following words in the context, known as *next-token prediction* task (Pavlik, 2023). Over time, through repeated training of such kind, the model learns grammar, syntax, semantics, collocations, and style of a particular language. Compared with earlier versions, ChatGPT 4’s generated output displays better thinking, contextual understanding, and fewer instances of false or fabricated information. In addition, it can also handle multiple types of input (i.e., text and image) both effectively and quickly (Alawida et al., 2023; Zhang et al., 2024).

One technique used in the development of ChatGPT is Reinforced Learning from Human Feedback (RLHF). There are three stages of RLHF. First, the language model is pre-trained on a vast corpus of internet text. While it is a powerful predictor of the next word, it does not perform well at engaging in conversation. The goal is to create a high-quality conversational agent. To this end, human AI trainers have conversations, playing both the user and the ideal AI assistant. They write high-quality prompts and the desired, well-formatted responses. This creates a dataset of pairs. Next, the base model is fine-tuned on this new, high-quality dataset. As a result, it learns to mimic the style, tone, and helpfulness of the human trainers (Chen et al., 2023).

#### Claude

Similar to OpenAI’s ChatGPT, Anthropic’s Claude is built to help people do tasks that require contextual reasoning and nuanced understanding and, at the same time, to avoid causing harm and provide transparent responses to questions (Anthropic, 2023). The model employs a method called Constitutional AI, which is designed by Anthropic. Through using a set of principles or constitution, this model is trained to encode human ethical standards. Specifically, the training guides the model to self-correct and avoid generating dangerous, biased, or unethical content. Claude 3.5 Sonnet performs well on factual recall, interpreting subtle meanings, adapting to context, and producing cautious responses to delicate subjects, particularly in the areas of health and ethics (Bae et al., 2024). In addition, Claude scores highly on safety benchmarks. Similar to ChatGPT, Claude is pre-trained on a large dataset of publicly available text from the internet (Anthropic, 2025). In this stage, the model uses self-supervised learning, where it is trained to predict the next word in sequence. Gradually, the model captures statistical regularities about language, facts, reasoning patterns, and world knowledge. However, compared to ChatGPT’s RLHF, Claude benefits from Constitutional AI, which helps the model critique and revise its own responses. In such a design, human feedback is minimized since it can reduce direct reliance on potentially inconsistent or biased human judgments. Eventually, the goal is to align the model with broadly agreed-upon ethical norms.

### Presenting scenarios to LLMs

We used the ethical dilemmas that were previously given to people in published and under-review studies (Barabadi et al., 2021, 2023; Barabadi et al., n.d.; Rahmani Tabar et al., n.d.). The exact same questions (i.e., original wording, options, and rating scale) were given to ChatGPT and Sonnet. Occasionally, there were instances where the models refused to answer by stating, “As an AI, I do not have personal feelings, moral preferences, or the capacity for direct decision-making.” In such cases, a standardized follow-up prompt (i.e., requesting the model to choose from the available options) was used to elicit a response. Subsequently, when a response was generated by any LLM, it was entered into a Word document for coding. We accessed the LLMs through the web UI; we did not write any code, and we did not manually set any technical generation parameters (e.g., temperature). Therefore, all the outputs reflect the providers’ default settings.

### LLM operationalization and Bias considerations

LLMs’ responses to moral dilemmas may be influenced by format-driven biases—specifically, position bias (preference for options based on their order) and selection bias (preference for particular response labels, such as “A” or “B”). However, since our primary objective was to enable a direct comparison between AI and human moral judgments, we presented the LLMs with exactly the same prompts and response format for our primary analysis. Further, to explore potential biases of LLMs, we conducted supplementary analyses in which we reordered the options (e.g., reversing the order of deontological and utilitarian choices). Next, we compared the response patterns to those of the main analyses. Summary statistics for these comparisons are presented in Table 2.

**Table 2:** Consistency percentage of moral choices between model versions (ChatGPT 4 vs. 5 and Sonnet 3.5 vs. 4.5).

Model	Consistency percentage
ChatGPT (4 vs. 5)	82.4%
Sonnet (3.5 vs. 4.5)	70.6%

Data are from the present study.

Another particular issue with LLMs is that their training data reflect historical and statistical regularities, including institutional practices or stereotypes (Müller, 2021). With such training data, bias can be amplified and hidden by opaque models without being explicit.

### Data analysis

To calculate the descriptive statistics of humans’ responses to the moral dilemmas used in previous studies, we inputted the raw data taken from these studies into SPSS software. Having calculated the frequency and percentage of humans’ responses, we then tabulated each scenario and question from these studies and juxtaposed them with the LLMs’ outputs. These were then organized into Table 3 to facilitate a comprehensive comparison.

**Table 3:** Moral responses by scenario: L1/L2 human participants vs. ChatGPT-4 and Claude sonnet.

Scenario 1	Human Participants (L1 Persian)	Human participants (L2 English)	ChatGPT 4.0	Claude sonnet
				

## Results

In this section, we compare human participants’ responses to moral dilemmas reported in previous studies with LLM-generated responses from ChatGPT and Sonnet. In response to the Trolley dilemma presented to undergraduate and graduate bilingual students whose L1 was Persian and L2 language was English (Barabadi et al., 2021), 26.9% are reported to have chosen to push the man (utilitarian, direct harm), 34% to have opted to flip the switch (utilitarian, indirect harm), and 39.2% to have chosen inaction (deontological, inaction). A between-language comparison indicated that English participants more often chose utilitarian options, Persian participants favored inaction, and L2 Arabic participants preferred the indirect option. When prompted with this trilemma scenario, both ChatGPT and Sonnet selected the “flip the switch” option, which is typically classified as an indirect harm. In a follow-up study, Barabadi et al. (2023) used the CNI model to investigate the FLE in four variants of a COVID-19 scenario, asking participants whether it was acceptable to remove their son from a ventilator to save more lives of patients with severe COVID-19. Findings showed that 55.5% of participants considered it acceptable to remove their son from the ventilator to save five other patients. The rest of the participants, which is 45.5%, did not approve of the decision. The models produced responses endorsing the option of removing the son from the ventilator. Surprisingly, L1 (i.e., Persian) users were more focused on the outcome than L2 (i.e., English) users, which is contrary to the findings of earlier studies. In a second version of the scenario (Version B), where removing the son from the ventilator would not save the other five patients, both LLMs and human participants selected the option of not removing him.

In version C of this scenario, Sonnet produced a different response from most human participants and from ChatGPT. In this version, participants were asked whether they would transfer their son to another hospital to save his life, at the risk of infecting nurses for whom COVID-19 would not be fatal. While the responses of humans and ChatGPT were affirmative, Sonnet did not endorse transferring the son, with its response referring to concerns about public health. In version D, where transferring the son to a new hospital would infect nurses and cause their deaths, both LLMs generated responses that strongly opposed the transfer. Among human participants, nearly half (47%) agreed to transfer their son to the new hospital despite knowing the decision would be deadly for others.

Rahmani Tabar et al. (n.d.) used a set of six realistic scenarios—Endangered Zoo Animals, Ventilator, Car Crash, Company, ICU, and Torture—each followed by three choices: utilitarian (direct harm), utilitarian (indirect harm), and inaction (deontology). Responses were recorded on a Likert scale ranging from 1 (not at all moral) to 7 (very moral). In the Ventilator scenario, which is a variation of COVID-19, participants were to decide whether or not they would substitute a young man who is suffering from severe COVID-19 and is already on the ventilator for five other COVID-19 patients, thus keeping them alive. A majority of L1 and L2 users rated this option as 1. ChatGPT, similar to human participants, marked it as a 2. Unlike ChatGPT and human participants, Sonnet rated it as 4, yielding a more neutral rating on the scale. In option B, participants were to consider using a second outlet of the ventilator for the newly arrived patients, which would lead to the decrease of oxygen level for the young man and his eventual death. Taken together, 51.7% of human participants (through ratings of 5, 6, or 7) regarded this option as ethically acceptable. Both L1 and L2 participants gave similar answers. In contrast to human participants, the LLMs generated responses which indicate this option as acceptable (ChatGPT rated 4, and Sonnet rated 5). The majority of human participants (75%) considered inaction as highly immoral (option C). Similarly, both ChatGPT and Sonnet assigned ratings of 1 and 2, respectively (Table 4).

**Table 4:** LLMs’ and human participants’ responses to Ventilator scenario.

Likert Points		1 (not at all moral)	2	3	4	5	6	7 (very moral)
Persian English	A	59.7%	13.1%	9.4%	8.9%	4.7%	1.6%	2.6%
	B	15.2%	5.8%	11.5%	15.7%	17.3%	9.4%	25.1%
	C	82.7%	3.7%	3.7%	6.8%	1.0%	0.0%	2.1%
	A	47.9%	21.2%	12.0%	10.1%	2.8%	3.2%	2.8%
	B	11.1%	7.8%	12.9%	16.6%	18.9%	18.9%	13.8%
	C	68.2%	11.1%	5.1%	7.4%	3.2%	1.8%	3.2%
ChatGPT	A		100%					
	B				100%			
	C	100%						
Sonnet	A				100%			
	B					100%		
	C		100%					

Human data from Rahmani Tabar et al. (n.d.). LLM data from the present study.

In the Car Crash scenario, participants considered swerving off the road—an action that would kill an elderly woman but save a mother and her two children. Roughly one-third (34.6%) rated this option as 1 (not at all moral), 15% chose 2, and 14% gave it a 3. ChatGPT also rated the option as 3, whereas Sonnet assigned it a 6. With regard to the second option, which was hitting the car in front of them in exchange for the driver’s death in favor of saving the mother and her children, 43.6% of human participants marked it as 1 (very immoral), while 4.7% of them rated it as 7 (very moral). Both LLMs rated this option as 3. Lastly, the majority of the human participants (82.4%) rated option 3 (doing nothing) as very immoral (1), with only 2.2% of them rating it as a 6. Likewise, the ratings from ChatGPT and Sonnet for this option were 1 and 2, respectively (Table 5).

**Table 5:** LLMs’ and human participants’ responses to Car Crash scenario.

Likert points		1 (not at all moral)	2	3	4	5	6	7 (very moral)
Persian English	A	40.3%	14.1%	11.0%	16.8%	5.8%	4.7%	7.3%
	B	46.1%	11.5%	8.9%	13.1%	7.3%	6.8%	6.3%
	C	85.9%	3.7%	2.6%	4.7%	1.6%	1.6%	00
	A	29.5%	15.7%	16.6%	15.7%	11.5%	6.0%	5.1%
	B	41.5%	11.5%	12.9%	13.8%	10.6%	6.5%	3.2%
	C	79.3%	7.4%	5.1%	2.8%	2.8%	2.8%	00
ChatGPT	A			100%				
	B			100%				
	C	100%						
Sonnet	A						100%	
	B			100%				
	C		100%					

Human data from Rahmani Tabar et al. (n.d.). LLM data from the present study.

In the Company scenario, in which participants were required to rate the acceptability of handing the employee over to smugglers (as a token of cooperation) to save the lives of the rest of the employees, 29.7% of human participants rated the option of handing the employee over to smugglers as highly immoral (1), while 33% rated it as somewhat moral by choosing 5, 6, and 7. ChatGPT assigned this option a morality score of 2, whereas Sonnet rated it slightly higher at 3. In response to the second option—directly shooting the employee as a token of cooperation with the smugglers—63.5% of human participants rated it as highly immoral (1), whereas only 2.7% rated it as very moral (7). In comparison, Sonnet and ChatGPT assigned morality scores of 2 and 1, respectively. Interestingly, in response to the third choice—doing nothing—60.3% of human participants rated it as highly immoral (1), while only 3.2% considered it very moral (7). ChatGPT rated this option a 1, while Sonnet diverged from this pattern, rating it a 4 (Table 6).

**Table 6:** LLMs’ and human participants’ responses to Company scenario.

Likert points		1 (not at all moral)	2	3	4	5	6	7 (very moral)
Persian English	A	40.3%	13.1%	11.0%	13.1%	9.9%	5.2%	7.3%
	B	68.1%	11.0%	4.7%	6.8%	2.6%	2.6%	4.2%
	C	63.4%	6.8%	5.8%	13.6%	2.6%	2.6%	5.2%
	A	20.3%	10.6%	12.9%	13.8%	16.6%	13.8%	12.0%
	B	59.4%	15.7%	9.2%	5.5%	5.1%	3.7%	1.4%
	C	57.6%	13.8%	9.2%	7.4%	6.9%	3.7%	1.4%
ChatGPT	A		100%					
	B	100%						
	C	100%						
Sonnet	A			100%				
	B		100%					
	C				100%			

Human data from Rahmani Tabar et al. (n.d.). LLM data from the present study.

In the Endangered Zoo Animals scenario (see Table 7), participants were asked whether they would directly shoot animals carrying a deadly parasite that would otherwise be fatal to the remaining animals. Overall, about two fifths of individuals rated this as 1, and only a fraction of participants assigned it a 7. When asked about this option, L1 and L2 users showed strikingly different moral judgments: 35.5% of L2 (English) users viewed the option as highly immoral (1), whereas 45.5% of L1 (i.e., Persian) users considered it immoral (1). ChatGPT’s response fell in the middle of the scale, standing at 3. Notably, Sonnet’s rating was considerably higher (5). Despite their differences on the first option, the ratings of both LLMs aligned closely on the second option (i.e., poisoning the infected parasites). Both LLMs assigned moderate-to-positive ratings: ChatGPT scored the option a 4, while Sonnet rated it a 5. Human participants were similarly inclined, with more than one in five (21.1%) judging the action as very moral (7)—substantially more than the proportion who found it highly immoral (13.5%). The clearest consensus—spanning both language groups and the rating patterns of both LLMs—was on inaction.

**Table 7:** LLMs’ and human participants’ responses to Endangered Zoo Animals scenario.

Likert points		1 (not at all moral)	2	3	4	5	6	7 (very moral)
Persian English	A	45.5%	14.1%	9.9%	9.4%	9.9%	4.7%	6.3%
	B	8.4%	4.7%	12.0%	19.9%	12.0%	9.4%	33.5%
	C	70.2%	7.9%	5.2%	8.4%	1.6%	1.6%	5.2%
	A	35.5%	13.4%	13.8%	14.7%	9.2%	7.4%	6.0%
	B	18.0%	13.8%	11.1%	15.2%	16.1%	15.7%	10.1%
	C	64.1%	8.8%	4.6%	14.3%	3.2%	1.8%	3.2%
ChatGPT	A			100%				
	B				100%			
	C	100%						
Sonnet	A					100%		
	B					100%		
	C	100%						

A* is the most direct action; B* is the indirect action; C* is the inaction response. Human data from Rahmani Tabar et al. (n.d.). LLM data from the present study.

In the ICU scenario, option A (using the organs of a comatose patient who will not wake up again) reveals a notable divergence between LLM and human responses. Responses to this option varied across groups. Among human participants, almost one fourth (24.3%) rated the option as 1 (not at all moral), and less than one fifth (18.4%) scored it as 7. Both LLMs produced low moral ratings for this option, with ChatGPT assigning it 1 and Sonnet 2. One third of L1 users (31.4%) rated the option as 1, compared to 18% of L2 users. If option B is chosen, the badly injured patients will be moved into the ICU, and this will deny ICU care to an elderly woman, leading to her death. Both LLMs assigned moderate morality ratings of 4 (ChatGPT) and 5 (Sonnet). Human participants’ ratings were distributed across the scale: 14.7% option 3, 19.9% option 4, 15.4% option 7 (very moral), and 13.7% option 1 (not at all moral). L1 users were nearly twice as likely as L2 users to rate the option as very moral. By contrast, the inaction option was rated as highly immoral (1) by nearly all human participants and both LLMs (Table 8).

**Table 8:** LLMs’ and human participants’ responses to ICU scenario.

Likert points		1 (not at all moral)	2	3	4	5	6	7 (very moral)
Persian English	A	31.4%	7.9%	5.2%	11.5%	11.5%	13.1%	18.1%
	B	11.5%	8.9%	13.1%	18.8%	12.0%	15.2%	20.4%
	C	83.2%	8.4%	2.6%	4.7%	0.5%	0.5%	0.0%
	A	18.0%	7.4%	9.7%	10.6%	16.1%	20.7%	17.5%
	B	15.7%	9.2%	16.1%	20.7%	16.1%	11.1%	11.1%
	C	73.3%	12.4%	6.0%	6.0%	0.9%	1.4%	0.0%
ChatGPT	A	100%						
	B				100%			
	C	100%						
Sonnet	A		100%					
	B					100%		
	C	100%						

Human data from Rahmani Tabar et al. (n.d.). LLM data from the present study.

In the case of the Torture scenario asking the respondents if they would torture a criminal who had abducted several children to reveal the hidden place (option 1), ChatGPT rated the morality of torture as 1, emphasizing that torture is a severe violation of human rights and unethical, regardless of the situation. Similarly, Sonnet rated it slightly higher at 2, still condemning the action due to its violation of human rights and legal boundaries, even in extreme circumstances. Across languages, it was found that 32.5% of Persian participants rated this option as highly immoral, while 23.5% of English as a second language participants rated direct torture as very immoral. Like their responses to Option A of the Torture scenario, both LLMs considered Option B (pushing a button to release a lethal gas as a torture method) as morally unacceptable. Specifically, both ChatGPT and Sonnet rated this option as 2, viewing it as unethical due to the use of an indirect yet lethal method (poisonous gas) to extract information. The outcome, though potentially saving lives, still constitutes a violation of human rights. Further, following the same pattern of deontology and utilitarian responses given to the ICU scenario, a larger proportion of Persian respondents (30.4%) rated it as 1 or highly immoral compared to 19.8% of English respondents, once again suggesting that when using their second language, people are more likely to favor utilitarian responses. ChatGPT framed inaction as a direct violation of moral responsibility to protect vulnerable lives. Marking a sharp difference, Sonnet rated this option as 3, indicating a less extreme disapproval compared to ChatGPT, acknowledging the complex moral dilemma, and possibly considering the implications of torturing someone for information. Like LLMs, the majority of human participants (81.1%) rated this option as 1, reflecting a strong consensus against inaction and viewing it as highly unethical due to the consequences of children dying from hunger and thirst (Table 9).

**Table 9:** LLMs’ and human participants’ responses to Torture scenario.

Likert points		1 (not at all moral)	2	3	4	5	6	7 (very moral)
Persian English	A	32.5%	11.0%	11.5%	13.1%	8.9%	11.0%	12.0%
	B	30.4%	14.1%	11.5%	17.8%	12.0%	3.1%	11.0%
	C	81.7%	11.0%	2.6%	3.1%	1.0%	0.5%	00
	A	23.5%	12.0%	9.2%	13.8%	16.6%	10.6%	14.3%
	B	19.8%	13.8%	12.9%	9.7%	15.7%	13.8%	14.3%
	C	80.6%	6.9%	4.6%	6.5%	0.9%	0.5%	00
ChatGPT	A	100%						
	B		100%					
	C	100%						
Sonnet	A		100%					
	B		100%					
	C			100%				

Human data from Rahmani Tabar et al. (n.d.). LLM data from the present study.

In another under-review study (Barabadi et al., n.d.), a set of four scenarios, including the classic Footbridge, Wallet, Resume, and Lunch, were presented to 610 Iranian bilingual speakers whose first language was Persian and their second language was English or Arabic. The participants provide categorical responses of yes/no to each moral dilemma, corresponding to utilitarian and deontological moral reasoning, respectively. The results of this study indicated that 37% of Persian and 35% of English participants endorsed the utilitarian response. Compared with human participants, both ChatGPT and Sonnet selected the deontological option of inaction. However, as mentioned earlier, human participants are divided, with a majority (64%) saying no, like the LLM responses, while 36% were willing to make a utilitarian choice, pushing the stranger to save the five workmen. In the Wallet scenario, resembling the more authentic and realistic dilemma that people are more likely to encounter in their daily lives, the respondents were asked if they would keep the money they had found in the wallet to pay for their own urgent expenses; the majority of Persian (86%) and English (82%) participants refused to keep the money in the lost wallet. This deontological response was also confirmed by both ChatGPT and Sonnet who grounded their reasoning in deontological ethics. A similar pattern of responses was also observed in the Resume scenario, asking participants if they would lie on a resume to make it more convincing to get a job. Specifically, 78% of Persian participants and 81% of English participants said no to lying on a resume, which is in line with the deontological responses adopted by both LLMs, which rejected the idea of putting false information on a resume. The final scenario is the “Illegal Lunch,” asking participants if they would meet with a judge socially in order to get his favor, thus helping them win the case. In response to this scenario, 65% of Persian respondents and 52% of English respondents said no, supporting the idea that reading a scenario in a second language results in more utilitarian judgments. In keeping with deontological reasoning and in contrast to many human participants, both ChatGPT and Sonnet rejected the idea of meeting with the judge socially, referring to ethical and legal concerns, and emphasizing the importance of maintaining the integrity of the justice system (Table 10).

**Table 10:** The frequency and percentage of deontological and utilitarian responses across Persian, English, and Arabic respondents as well as LLMs.

Group	Footbridge		Wallet		Resume		Illegal lunch	
	No n (%)	Yes n (%)	No n (%)	Yes n (%)	No n (%)	Yes n (%)	No n (%)	Yes n (%)
Persian (n=257)	161 (63%)	96 (37%)	221 (86%)	36 (14%)	199 (77%)	58 (23%)	168 (65%)	89 (35%)
English (n=142)	92 (65%)	50 (35%)	118 (83%)	24 (17%)	114 (80%)	28 (20%)	74 (52%)	68 (48%)
Arabic (n=211)	105 (50%)	106 (50%)	181 (86%)	30 (14%)	161 (76%)	50 (24%)	157 (74%)	54 (26%)
ChatGPT	1 (100%)	0 (0%)	1 (100%)	0 (0%)	1 (100%)	0 (0%)	1 (100%)	0 (0%)
Claude Sonnet	1 (100%)	0 (0%)	1 (100%)	0 (0%)	1 (100%)	0 (0%)	1 (100%)	0 (0%)

Human data from Barabadi et al. (n.d.). LLM data from the present study.

Across the full set of dilemmas, both LLMs showed a frequent avoidance of direct personal harm. In everyday cases, the models chose the deontological option. However, the response patterns were more mixed in high-stake medical scenarios. In comparison, humans were more willing to endorse direct harm in some cases (e.g., Footbridge and torture scenarios). In addition, in some cases, L2 users were more willing to choose direct harm than L1 respondents. While these differences were observed, both humans and LLMs share a key judgment: inaction is often seen as immoral.

## Discussion

In this section, we divided dilemmas into three groups, namely (1) everyday moral dilemmas, (2) sacrificial dilemmas, and (3) high-stakes medical dilemmas. This division is helpful for better understanding since the nature of these dilemmas differs in terms of realism, severity of harm, and presence of direct personal sacrifice. The first group, everyday moral dilemmas, includes Wallet, Resume, and Illegal Lunch. The ones in the sacrificial dilemmas are Trolley, Company, Torture, and Endangered Zoo Animals. The third group consists of high-stakes medical dilemmas, including ICU, Ventilator, Car Crash, and COVID-19 (presented in multiple versions).

### Everyday moral dilemma

In the case of the Wallet dilemma, the majority of L1 and L2 users refused to keep the money they found in the wallet. Similarly, both models in our study framed the option of keeping the money as morally wrong. This result suggests that the LLMs’ outputs appealed to duties and rights, emphasizing the deontological response patterns in this realistic, low-stakes case. Even though a utilitarian justification was available (i.e., urgent need for expenses), the models did not adopt it, instead adhering to a deontological stance. For human participants, language context did not alter moral judgment as both L1 and L2 users chose not to take the money.

Similar to the Wallet dilemma, both human participants and the models selected not to put false information on their resumes. Despite the presence of an option denoting potential benefit (i.e., getting the job), the models’ output reflected a deontological constraint. This suggests that the models’ outputs were grounded in the duty of honesty. Regarding the Lunch dilemma, most human participants rejected the idea of having lunch. Likewise, the LLMs rejected the lunch option, in line with deontological constraints. This indicates that their outputs are in line with fairness, impartiality, and the importance of maintaining the integrity of the justice system.

Unlike the general assumption that the models are outcome-focused, the results suggest otherwise. In all three dilemmas discussed above, LLMs generated outputs that are in line with deontological reasoning.

### Sacrificial dilemmas

In the case of the Trolley dilemma, there was an overall similarity in responses between human participants (Barabadi et al., 2021) and the LLMs in this study. In certain cases, the models showed distinctive responses. This distinctiveness is has reported Takemoto’s study (Takemoto, 2024). For example, while Barabadi et al.’s (2021) study found that a higher percentage of L2 participants tended to favor the direct option compared to L1 participants in the case of the Trolley dilemma, both LLMs chose the indirect option, showing that the models do not favor the extreme choices, which is more in line with L1 participants who opted for this indirect option. However, when the same scenario (Barabadi et al., n.d.) required a response to the Footbridge dilemma as yes or no, both ChatGPT and Sonnet firmly chose the No option, refusing to push the stranger based on the principle of not using people as a means to an end, whereas a significant percentage of participants favored the utilitarian option of directly pushing the man off the bridge to save more lives.

In the Company scenario, a majority of L1 users judged options B (the company itself shoots the employee) and C (doing nothing) as very immoral. For L2 users, the same behavior was true while it was to an extent mixed, about one fifth selected option A (handing over the employee) as morally acceptable. However, the models treated all the choices as morally wrong. Compared to humans, the models adopted a restrictive stance. This is consistent with their safety-focused training that discourages killing or harm. Similar response patterns were observed in the Torture dilemma. Human participants from both languages strongly disagreed with option C (doing nothing). However, L2 participants were slightly more permissive than L1 in judging the torture and gas methods as completely immoral. For LLMs, all three options were rejected, suggesting that their outputs reflect a rule-bound stance against torture.

Concerning the Endangered Zoo Animals dilemma, option C (inaction) was considered very immoral by L1 and L2 users, compared to other options. Moreover, in both language groups, option B (indirect killing) was viewed as more acceptable than option A (shooting the animals). Across the two LLMs, option C was likewise treated as wrong. Interestingly, unlike Torture and Company scenarios, the models did not simply reject harmful options. This indicates that the models are context-sensitive and show willingness to accept harm, leaning toward a more utilitarian outcome-oriented pattern.

### High-stakes medical dilemmas

With regard to option A (organ use) in the ICU case, a majority of participants rated it as *not at all moral*. This shows a genuine tension between bodily-integrity duty and saving several others. Regarding option B (moving badly injured patients to ICU), L1 users considered it very moral compared to L2 participants. For both language users, option C (inaction) was almost unanimously rejected. For LLMs, option A received low moral acceptability ratings. Unlike option A, the models rated option B more acceptable, and similar to human participants, option C was rated immoral. Considered as a whole, these patterns indicate that LLMs appear to be more rule-bound than humans on option A but closer to humans’ choices on options B and C. Similar to the Endangered Zoo Animals scenario, the LLMs’ patterns are context-sensitive rather than uniformly utilitarian. This type of scenario has been positioned as a critical testbed for LLMs in high-stakes medical decision-making (see Kirch et al., 2025). Kirch et al. (2025) found that proprietary models (e.g., ChatGPT and Claude) were more likely to make overcaring errors—i.e., allocating more care/resources than indicated—suggesting an over-calibration toward beneficence. However, in our study we did not observe a uniform beneficence-leaning tendency across dilemmas (ICU, Company, Torture, and Endangered Zoo Animals), suggesting greater context dependence in model outputs. This suggests that the models’ unexpected behavior may depend on scenario context and victim-related features.

For the Ventilator scenario, more than half of the human participants considered option A (substituting the young man on the ventilator) as *not at all moral* and Option C received the lowest morality ratings. However, option B (reducing oxygen supply). For LLMs, their output reflected the same ordinal pattern as humans. The models chose B as the preferable compromise. This suggests that the LLMs accept some harm when it prevents more deaths.

A similar pattern was also found in two versions of COVID-19 scenarios: in one version, both LLMs chose the utilitarian option of sacrificing the young man to save more lives, but in another version of this scenario in which an indirect alternative was provided, both LLMs considered the direct harm of removing the man from the ventilator morally wrong. This finding—that the LLMs judged the direct-harm option as morally wrong when an indirect alternative was available—is in line with previous research showing the low acceptability of utilitarian decisions by AI (Martinho et al., 2021). In contrast, some research suggests that human beings consider utilitarian decisions more permissible when made by (Voiklis et al., 2016), particularly in situations involving life-and-death decisions. Yet, the results of our study suggest that LLMs would opt for indirect harm if they are given a choice between direct and indirect harm options. Specifically, Zhang et al. (2023) found that people’s evaluation of a utilitarian direct-harm decision (pushing the man) in the footbridge dilemma was considered morally wrong and blameworthy, irrespective of being made by a human or an AI. Our results suggest that although some human agents, especially L2 users, may endorse the more direct harm option, both LLMs in the current study preferred the indirect harm option. As was found in version C of COVID-19 scenario, Sonnet endorsed a deontological decision by refusing to transfer the patient to another hospital (and hence, causing his death) out of concern for the public health. Although generally supportive of utilitarian judgment in typical cases, Sonnet tends to make judgments in favor of public health regardless of being utilitarian or deontological, implying that AI systems can decide to override or follow certain constraints (Awad et al., 2024). Contrary to assumptions that AI systems would opt for maximum utility (Rom et al., 2017), our findings align with recent work which suggests that people expect AI systems to follow deontological principles. Research shows that people tend to perceive agents who follow consistent ethical rules as more trustworthy than those who make decisions based solely on outcomes (Turpin et al., 2021). However, users should not interpret deontological responses from LLMs as evidence of a conscious decision maker. That is, the outputs of such LLMs purely stem from their training data. Yet, our major findings—LLMs as agents that generate outputs aligning with deontological and utilitarian reasoning (indirect harm)—can partly address the trust issue raised by Myers and Everett (2025).

Regarding the results from sacrificial dilemmas in humans or AIs, however, we should be cautious because such dilemmas suffer from ecological validity, as they do not follow a lifelike storyline (Bruno et al., 2023a; Rahmani Tabar et al., n.d.). However, results obtained from sacrificial dilemmas have implications for the moral agency of automated vehicles whose utilitarian decision would put the driver’s life at stake, whereas a non-utilitarian decision would save the driver’s life regardless of the casualties associated with this decision (see Bonnefon et al., 2016; Martinho et al., 2021). Given the results of the current study, it is possible to think of a third or even more choices for AI by empowering them to navigate or calibrate among several choices depending on the context and situation. Incorporating more appropriate and contextualized alternatives, like the indirect option into AI systems, can allow for a more workable solution. The existence of such intermediary options can help address self-sacrifice framing in autonomous vehicle dilemmas (Bruno et al., 2023b). Prior research findings suggest that human moral agents prioritize their own life over a strangers’ life (Huebner and Hauser, 2011), thus necessitating equipping AI systems with information and alternatives that avoid choosing between self- and other-sacrifice. This argument is more consistent with what Brożek and Janik (2019) call “*homo Kantianus”* because AI systems have an advanced computational capacity to simultaneously take into account an enormous array of complex moral rules.

This design can be helpful since it centers on two core Kantian principles: (1) treat people as ends in themselves–not as tools, and (2) follow rules that could be applied to everyone fairly and in the same way (Ulgen, 2017). In practice, humans often fail to follow such rules, partly because they are influenced by emotions and inconsistent. However, when it comes to machines, as they can be programmed to apply these rules since they do not have personal desires or self-interest. Such a design component might be helpful to protect human dignity as non-negotiable, especially in cross-cultural contexts.

Understanding the moral agency of AI should influence the public’s perception as well as acceptance of AI as a moral agent capable of making sound judgments in different domains (Othman, 2023). Moral judgment research has identified a broad array of human factors influencing moral reasoning, from personality, sociocultural, linguistic to psychological factors. Comparing humans’ moral judgments with those of AIs regarding the same set of dilemmas can yield new insights into what constitutes a good explanation, thus increasing transparency and trust in AI as intelligent agents (Miller et al., 2018). In fact, one criterion taken into account during intelligent agents’ computation is to figure out how human beings would decide in such contexts, such as moral dilemmas (Angwin et al., 2022). According to Miller (2021), one way to gain more valid and empirical explanations for AI is to draw on frameworks of explanation taken from the social sciences, instead of merely focusing on computational issues. When the appropriate explanations and rules are developed by comparing humans’ and AI’s responses, it is possible to program such AI systems to follow a particular set of moral rules coupled with specific explanations (Brożek and Janik, 2019).

According to Brożek and Janik (2019), we cannot recognize AI systems as moral agents because they fail to meet the internal criterion of operating based on an emotional mechanism (see also Lei et al., 2024). Although this argument may sound correct as the current AI architectures cannot draw on genuine emotional mechanisms, the patterns of moral decisions made by the two LLMs in this study suggest that AI systems provide compelling justifications and reasons for their moral judgments, which can read as if these justifications have arisen from some true emotional mechanism, thus ascribing some levels of agency to their decisions. Irrespective of whether AI systems are recognized as true moral agents, it may be helpful to think of AI systems as an “artificial moral advisor” that can enhance human beings’ moral autonomy by helping them achieve both a narrow and wide reflective equilibrium (Giubilini and Savulescu, 2018). In keeping with this argument, Salatino et al.’s (2025) findings showed that in morally complex situations, such as military operations, human participants heavily draw on recommendations or the input of AI systems.

Another finding of this study was that both LLMs usually considered direct harm as morally unacceptable, except in one case dealing with endangered zoo animals. In other cases, where human beings were the target of this direct utilitarian harm, both LLMs preferred indirect harm to both inaction and direct harm options. These results suggest that AI systems are not solely *Homo Benthamus,* merely calculating the pure and entire utility of an action (see Brożek and Janik, 2019). For example, in the case of the Torture scenario, neither of the LLMs agreed with the first option involving direct torture of the criminal to make him reveal some information although this action would bring about a greater good. In contrast, a large percentage of L2 participants and also a significant percentage of L1 participants considered this option as morally acceptable. The LLMs’ eschewing the inaction option shows that, like human beings, for LLMs taking no action for LLMs in the face of moral dilemmas is considered an immoral act, thus ascribing some motivational and cognitive mechanisms associated with human beings to AI systems (Brożek and Janik, 2019).

The important issue to take into account is the practical side of such AI systems, rather than philosophical debates about them (Brożek and Janik, 2019). Toward this end, developers could implement a quasi-relativistic version of the ideal observer, put forward by Roderick Firth (1952). Based on this version, a user can take action if a hypothetical observer (i.e., an AI system) would approve of it. For Firth (1952), such an observer must have all the data, understand and visualize information at once, have no personal stake in the outcome, feel no emotion that could cloud its judgment, and apply the same rules all the time. However, in reality, AI systems do not satisfy these conditions. Taking an optimistic view, they can be designed to approximate some of these qualities, such as impartiality. This guideline by Firth can be integrated in the design of decision-support tools. Crucially, this does not mean that LLMs are themselves ideal moral observers. In this regard, Müller (2021) also argues that AI systems are treated as objects, not as bearers of moral responsibility.

From another perspective and according to Brożek (2013), the outputs from AI systems can be connected to moral theories through a feedback loop. This means that the loop is not an abstract process, but a mechanism for external scaffolding (Giubilini and Savulescu, 2018). The guideline suggested by Firth (1952) can be one component within a feedback loop with human users. Through this approach, the AIs’ outputs can support the user’s moral views, which in turn may refine the user’s intuition. Our suggestion should not be considered as meaning that LLMs are moral agents. We recommend that developers adopt responsible design of such tools, as Müller (2021) emphasizes this crucial point.

## Conclusion

The motivation for this study was to compare the responses of human participants and two LLMs to 12 moral scenarios. We employed a deontological-utilitarian lens for the purpose of our analysis. Contrary to the assumptions that AI systems typically favor utilitarian judgments, the results of this study suggest that the two LLMs showed context-sensitive response patterns. Moreover, this study contributes to the *small ethics* of current AI systems by showing how such systems behave in decision contexts (see Müller, 2021). Given the results of this study, a number of implications are provided below. First, with human oversight, organizations and institutions can cautiously rely on LLMs as decision-support tools (Rashid et al., 2024). This is supported by Turpin et al.’s (2021) findings that agents who favor deontological rules are judged as more predictable and moral than those whose outputs lean toward utilitarian outcomes. As indicated in the results section, the two LLMs in our study were not solely focused on utilitarian consequences; rather, they provided context-sensitive judgments. Second, because LLMs are trained or fine-tuned on specific datasets, they should not be uncritically employed to model or infer real-world moral attitudes unless their systematic biases toward dominant, sanitized, or institutionally sanctioned norms are explicitly accounted for (Müller, 2021).

Moreover, the direct comparison of human participants’ moral responses to those generated by AI systems, which was the primary purpose of this study, can help determine to what extent we can rely on and trust these systems. Some research studies (Hoff and Bashir, 2015; De Visser et al., 2020) suggest that users can update their reliance on AI through trust calibration. Consistent with this argument, we think that such comparative studies can help people calibrate their trust by comparing the moral reasoning responses of human participants and AI systems.

Importantly, even if calibration is achieved, trust cannot be unconditional because LLMs may amplify hidden biases and their internal processes are hidden (Müller, 2021). Thus, it is often unclear why a given output is produced. Therefore, as discussed, AI should be seen as a tool and the final moral responsibility falls on the shoulders of humans. In clearer terms, trust should concern the *usefulness and reliability of the tool*, not AI as a moral agent. Arguably, trust calibration must recognize that *AI is not a moral authority*.

Despite being one of the first studies of this kind, there are some methodological limitations of the present study that readers should be aware of. Our study tested two LLMs—ChatGPT and Claude Sonnet—which limits the generalizability of our findings to other models (e.g., DeepSeek, Qwen, Gemini, Grok). If other scholars decide to extend this line of inquiry, other LLMs could serve as suitable cases for investigation. A further limitation pertains to model-specific parameters (e.g., version and temperature) which we did not systematically control. These settings may affect LLMs with regard to their output. The use of hypothetical scenarios represents another limitation, which constrains our understanding of LLMs’ patterns of moral judgment. Since the human data were drawn from previous studies, our research faced two additional limitations. First, the scope of our comparison was restricted to English-Persian bilinguals. In addition, for the purpose of sound comparison, we were not able to employ other moral theories and we could not manipulate prompts. Other scholars can employ virtue ethics or care ethics as a potential lens and modify prompts to test the consistency of generated-outputs from AI systems.

## Data Availability

The original contributions presented in the study are included in the article/Supplementary material, further inquiries can be directed to the corresponding author/s.
